# Cutis Marmorata

**Published:** 1910-02-12

**Authors:** 


### CUTIS MARMORATA.

Everyone is familiar with the marbled mottling which may appear on a healthy skin upon sudden exposure to the cold air. In certain subjects, particularly children and young persons who suffer from cold hands and feet, this condition has been called chilblain circulation. So-called chilblain circulation is particularly common in those who have a tendency to benign tuberculous lesions, such as tuberculous glands, and in those who suffer from tuberculides or from lupus erythematosus. In these subjects "cutis marmorata," or, as it is sometimes called, "livedo annularis," is often well marked on exposure to cold. The forearms, the inner sides of the thighs and legs, and especially the neighbourhood of the knees, are the parts where it is generally most evident. Here we see a dusky red or purplish mottling or network enclosing pale areas of normal-coloured skin. There is no change in the epidermis, and the colour fades on pressure or when the part gets warm. Sometimes mottling is present also upon the trunk. Its clinical interest lies in the fact that it may be mistaken for the macular erythema of the exanthem stage of syphilis, but a little care will always enable one to distinguish it. Whereas the mottling of livedo is in the form of a network enclosing pale areas, the macular rash of syphilis is exactly the reverse, namely, red areas separated by a paler network of normal skin. The explanation of this inverse arrangement of the mottling from cold, and the macules of roseola is afforded by the anatomical arrangement of the blood-vessels of the skin. It has been demonstrated by means of incomplete injections that there exist in the skin circumscribed rounded areas where the blood-flow is more active, while at the margin of these areas the circulation is sluggish. The central part of the areas of active circulation is supplied by a large artery which sends branches outwards to meet the venous circulation at the margin. Thus it happens that any active disturbance of the circulation of the skin may lead to the formation of erythematous patches, while a passive congestion determines a stagnation of venous blood at the margins of these areas. An analogous condition is seen in post-mortem lividity, where, owing to the presence of fluid blood in the veins which exudes through their walls, a dark network-like mottling is produced. There have also been described cases in which this condition of cutis marmorata occurs in the living subject as a permanent condition. The ætiology of these cases is not known, but the skin trouble consists in a dull red mottling in the form of a network about the knees, thighs, and legs, which is persistent and which does not fade entirely on pressure, but leaves a pigment stain behind. A similar condition is also well known as the result of constant exposure to heat, as in sitting with legs close to the fire. In these cases, which have been called "erythema ab igne," the mottling results in deep pigmentation which is very persistent.

